# Health care workers’ experiences of calling-for-help when taking care of critically ill patients in hospitals in Tanzania and Kenya

**DOI:** 10.1186/s12913-024-11254-y

**Published:** 2024-07-17

**Authors:** Elibariki Godfrey Mkumbo, Tamara Mulenga Willows, Onesmus Odongo Onyango, Karima Khalid, John Maiba, Carl Otto Schell, Jacquie Oliwa, Jacob McKnight, Tim Baker

**Affiliations:** 1 The Health Systems, Impact Evaluation and Policy Department, Ifakara Health Institute, Dar Es Salaam, Tanzania; 2 Health Systems Collaborative, University of Oxford/ Wolfson Institute of Population Health, Queen Mary’s University London, London, UK; 3 Health Services Unit, KEMRI-Wellcome Trust Research Programme, Nairobi, Kenya; 4 Department of Emergency Medicine, Muhimbili University of Health and Allied Sciences, Dar Es Salaam, Tanzania; 5 Department of Global Public Health, Karolinska Institutet, Stockholm, Sweden; 6 Centre for Clinical Research Sörmland, Uppsala University, Eskilstuna, Sweden; 7 Department of Medicine, Nyköping Hospital, Nyköping, Sweden; 8 Health Systems Collaborative, University of Oxford, Oxford, UK; 9 Department of Clinical Research, London School of Hygiene and Tropical Medicine, London, UK

**Keywords:** Calling for help, Critical illness, Critical care, Emergency care, Communication, Health workers

## Abstract

**Background:**

When caring for critically ill patients, health workers often need to ‘call-for-help’ to get assistance from colleagues in the hospital. Systems are required to facilitate calling-for-help and enable the timely provision of care for critically ill patients. Evidence around calling-for-help systems is mostly from high income countries and the state of calling-for-help in hospitals in Tanzania and Kenya has not been formally studied. This study aims to describe health workers’ experiences about calling-for-help when taking care of critically ill patients in hospitals in Tanzania and Kenya.

**Methods:**

Ten hospitals across Kenya and Tanzania were visited and in-depth interviews conducted with 30 health workers who had experience of caring for critically ill patients. The interviews were transcribed, translated and the data thematically analyzed.

**Results:**

The study identified three thematic areas concerning the systems for calling-for-help when taking care of critically ill patients: 1) Calling-for-help structures: there is lack of functioning structures for calling-for-help; 2) Calling-for-help processes: the calling-for-help processes are innovative and improvised; and 3) Calling-for-help outcomes: the help that is provided is not as requested.

**Conclusion:**

Calling-for-help when taking care of a critically ill patient is a necessary life-saving part of care, but health workers in Tanzanian and Kenyan hospitals experience a range of significant challenges. Hospitals lack functioning structures, processes for calling-for-help are improvised and help that is provided is not as requested. These challenges likely cause delays and decrease the quality of care, potentially resulting in unnecessary mortality and morbidity.

**Supplementary Information:**

The online version contains supplementary material available at 10.1186/s12913-024-11254-y.

## Introduction

During their stay in hospital, the condition of some patients [1] deteriorates and becomes life-threatening [2]. Such “critical illness” is very common, affecting an estimated 45 million adults each year [3]. Development of critical illness rarely occurs without warning, and is usually preceded by a series of often undetected changes in vital clinical signs over a period of hours [4]. Recognizing these changes and initiating a timely response is a healthcare priority and a key component of high-quality healthcare [5].

Care of critically ill patients can be challenging, and health workers often need assistance from other colleagues to provide the care. Their efforts to seek assistance is what we are terming, ‘calling-for-help’, in this paper, and refers specifically to the process of initiating communication and relaying the call to a colleague or colleagues. The desired outcome of calling-for-help is the receipt of the requested help. For health workers, calling-for-help is an essential part of initiating and escalating care for critically ill patients, getting more senior or advanced involvement and to successfully manage the patient’s condition [6]. Failure to provide appropriate care in hospitals may be due to a failure of the calling-for-help system [7] and effective calling-for-help is an important safety and quality-of-care issue that has a profound impact on patient care outcomes [8, 9].

Over the years, there has been notable attempts to improve calling-for-help systems [10]. A variety of intra-hospital alerting, and response systems have been put in place and hospitals have introduced communication systems in-part to enable calling-for-help [9]. Efforts to improve intra-hospital communication and calling-for-help systems can be traced back to the 1950s when the first electronic paging devices were introduced [11] and by 1980s, pagers had become popular in many countries. Later, cell phones replaced pagers and communication and alerting system innovations have been developed such as early warning systems (EWS), rapid response teams (RRT) and the ‘Situation, baseline, assessment and recommendation (SBAR)’ communication protocol [12] to improve the detection and care escalation of patients at risk of deterioration [13, 14].

Most evidence around calling-for-help systems come from high income countries, and to our knowledge the state of calling-for-help in hospitals in Tanzania and Kenya has not been the topic of any formal studies. We aim to describe health workers’ experiences about calling-for-help when taking care of critically ill patients in hospitals in Tanzania and Kenya.

## Methods

### Study design

We used a qualitative descriptive design to explore and describe the experiences of healthcare workers in Kenya and Tanzania regarding calling-for-help when caring for critically ill patients. This design has been recommended as well-suited for gaining a comprehensive understanding of a specific phenomenon [15, 16]. Data collection was part of the “Provision of Essential Treatment in Critical Illness” (POETIC) project investigating the care of critically ill patients in Kenya and Tanzania during the global COVID-19 pandemic.

### Setting

The study included five government-owned hospitals at the primary, secondary, and tertiary levels in Kenya and five in Tanzania. These hospitals provide a wide range of medical services including emergency departments, outpatient services, and inpatient care including pediatric care, general medical care, maternity care, and surgical care. The hospitals were selected based on pre-existing relationships with the hospitals and ease of access during the COVID-19 pandemic.

### Participants and recruitments

A purposive sampling method was used to identify participants in the hospitals. This method is recommended when exploring a phenomenon from a group of respondents who are likely to provide varying information [17]. Based on their experience in caring for critically ill patients in the designated hospital departments, nurses and physicians from intensive care units, emergency units, out-patient departments, pediatric departments, medical wards, maternity wards and labor wards were included. Initially, we established contact with the heads of the departments, who helped us identify potential participants. Prior to the interviews, the identified potential participants were approached by the researchers responsible for data collection (EM, JM, and OO). The researchers provided an explanation of the study’s purpose, procedures, privacy and confidentiality. Participants were informed about their right to end the interview at their convenience and were made aware of their right to withdraw from the study at any point, without any consequences or repercussions. Participation was voluntary, and participants were under no obligation to take part in the study. All information and consent discussions took place before the interview sessions, ensuring that participants had ample time to consider their involvement and raise any concerns. Participants were given chance to ask questions, and to check their understanding they were asked to repeat back to the researcher what they understood about participating in the research. All participants provided written consent for their participation, for interviews to be recorded, and for the anonymous use of quotes from the interviews. As we were purposefully looking for participants with experience in calling-for-help and as nurses are the main cadre of health workers calling-for-help, the majority of our participants were nurses. The number of participants was guided by thematic saturation in which additional data no longer led to any new emergent themes, as commonly used in qualitative research [18], and was expected to include 15 to 20 in each country [18].

### Data collection

Data collection took place between January and December 2021 using a semi-structured interview technique. We employed this technique as recommended in similar studies [19]. Researchers with qualitative research experience (EM, JM, and OO) conducted the interviews, which lasted between 45 and 60 min. The interviews were conducted in settings that prioritized participant comfort, privacy, and convenience. In Tanzania, the interviews were held physically in private and quiet spaces within the hospitals. In Kenya, due to COVID-19 restrictions, interviews were conducted virtually, using secure and confidential video conferencing platforms. Interviews were arranged at a time that best suited the participants and were assured that their responses would be anonymized, and any identifying information would be kept confidential. Participants were not paid.

### Data collection tool

Team of researchers experienced in qualitative research (EM, JM, and OO) developed an interview guide. The questions were designed to be open-ended, allowing participants to share their experiences and perspectives in depth. Before commencing the main data collection phase, the interview guide was pre-tested on a small group of healthcare workers who were not included in study sample. Feedback from the pretest was used to refine and modify the interview guide.

### Data management and analysis

All audio recordings of the interviews were transcribed verbatim by people fluent in English or Swahili, and Swahili transcripts were translated into English. To preserve participants’ worldviews, bilingual individuals with in-depth knowledge of the cultural terms and healthcare terminology in both languages conducted the translations. The translated transcripts were cross-checked by an experienced researcher, EM, by comparing interview notes, translations, and pre-data-coding completeness. Any discrepancies or loss of meaning during translation were identified and rectified through a collaborative effort among the research team. Validation of the data was performed by the researchers in the form of a feedback cross-check meeting of the findings with the health workers in the institutions, and discrepancies found were discussed and reconciled.

Transcripts were managed in NVivo-12 qualitative data software and read several times by EM and JM for familiarization. An inductive thematic approach was used to code, analyze and interpret the data using inductive analysis [20]. The choice of this approach was made to allow themes to emerge naturally from the data, rather than imposing preconceived categories [20], in assessing the calling -for-help quality of care by looking on structures,processes and outcomes we were guided by a modified Donabedian quality-of-care framework [21] Structures are the physical and organizational resources available to provide care, such as staffing levels, medicines and equipment. Processes are the actions taken to provide care to patients, such as observing vital signs and providing treatments. Outcomes are the results of the care provided, in this study the outcomes of the process of calling-for-help.

The data were organized into codes by one of two researchers with social science backgrounds (EM, JM), as per approach of Sarantakos et al. [22], where JM and EM coded transcripts independently and then reconciled by managing the discrepancy to arrive to final codes and codes organization was guided by inductive thematic approach. Consistency was ensured through random selection and analysis of some of the transcripts by the other researcher, and any discrepancies discussed, and standards set to guide the rest of the coding process. Similar codes were grouped into categories, and then themes were identified that were revised as new codes and categories emerged through the process. In the final phase, the results were refined through a review done by a clinical expert (TB) who considered the relevance and applicability of the identified themes to clinical settings.

## Results

A total of 30 health workers – 11 doctors and 19 nurses – in the 10 study hospitals across Kenya and Tanzania were interviewed (Table 1).

**Table 1:** Characteristics of participants

Country	Hospital Name	Hospital Level	Cadre	Department	Work Experience (years)	Education
**Kenya**	Hospital 1	Level 6 (tertiary)	Nurse	Emergency Department	10	Masters (MSc in Emergency Nursing)
			Nurse In-charge	Intensive Care Unit	16	Masters (MSc in Critical Crae Nursing)
			Nurse	Medical ward	15	Diploma (Kenya Registered Community Health Nurse)
			Nurse	Labor ward	7	Bachelor’s (BSc in Nursing)
	Hospital 2	Level 4 (secondary)	Nurse In-charge	Pediatric Outpatient	10	Diploma (Kenya Registered Community Health Nurse)
			Doctor	Maternity Ward	1.5	Bachelor of Medicine and Surgery (MBChB)
			Nurse (Nursing services manager	Administration	15	Bachelor’s (BSc in Nursing)
	Hospital 3	Level 5 (secondary)	Clinical officer (non-physician clinician)	Out-patient Department	2	Diploma in Clinical Medicine
			Clinical Office	Intensive Care Unit	5	Diploma in Clinical medicine
			Nursing services manager (NSM)	Administration	22as a nurse, 2 years as the NSM	Bachelor’s (BSc in Nursing)
	Hospital 4	Level 5 (secondary)	Doctor	Emergency Department	1	Bachelor of Medicine and Surgery (MBChB)
			Nurse In-charge	Emergency Department	3	Bachelor’s (BSc in Nursing)
	Hospital 5	Level 4 (secondary)	Clinical Officer (non-physician clinician)	Emergency/Out-patient Department	4	Diploma in Clinical Medicine
			Doctor	Emergency/Out-patient Department	2	Bachelor of Medicine and Surgery (MBChB)
			Nurse	Isolation Unit	8	Diploma (Kenya Registered Community Health Nurse—KRCHN)
**Tanzania**	Hospital 1	Regional Referral	Doctor-Emergency Medicine Specialist	Emergency Department	12	Master in Critical Care Medicine
			Doctor-Internal Medicine Specialist	Internal medicine department	16	Masters in Internal medicine
			Nurse	Emergency Department	15	Diploma in Nursing
	Hospital 2	District hospital	Doctor	Out-patient Department	17	Doctor of Medicine
			Nurse	Maternity Ward	14	Bachelor’s (BSc in Nursing)
			Nurse	Medical Ward	19	Diploma in nursing
	Hospital 3	Regional Referral	Doctor	Emergency Department	15	Master in Critical care medicine
			Nurse	Out-patient Department	13	Diploma in nursing
			Nurse	Surgical /Medical ward	9	Diploma in nursing
	Hospital 4	National Referral	Nurse	Intensive Care Unit	16	Bachelor of science in nursing
			Nurse	Emergency Department	15	Bachelor of science (Critical care nursing)
			Nurse	Pediatric Ward	23	Bachelor’s(Science Nursing)
	Hospital 5	District hospital	Doctor	Medical Ward	26	Doctor of Medicine
			Doctor	Maternity Ward	13	Doctor of Medicine
			Nurse	Outpatient Department	14	Diploma in nursing

### Summary of key findings

**Fig. 1:**
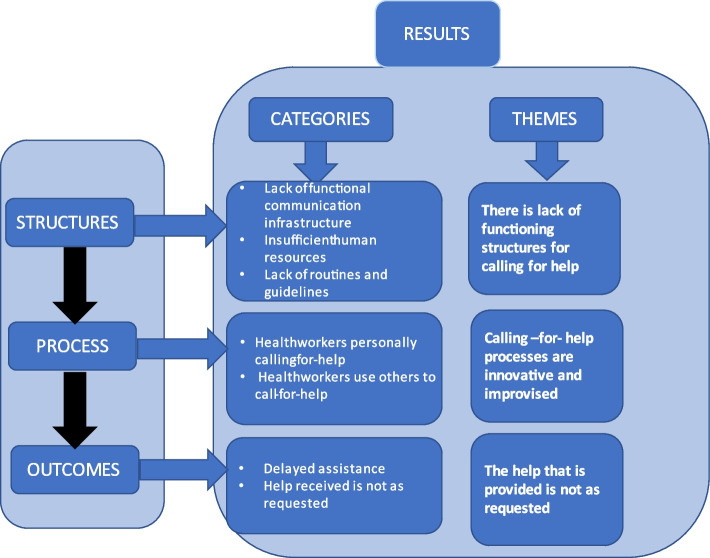
Summary of findings

Participants’ experiences with calling-for-help when taking care of critically ill patients can be organized into three thematic areas: 1) Calling-for-help structures: there is lack of functioning structures for calling-for-help; 2) Calling-for-help processes: the calling-for-help processes are innovative and improvised; and 3) Calling-for-help outcomes: the help that is provided is not as requested (Fig. 1).

#### Theme 1 Calling-for-help structures: there is a lack of functioning structures for calling-for-help

Participants described structural challenges impeding the smooth execution of calling-for-help. The structural challenges concerned a lack of communication infrastructure, insufficient human resource and a lack of routines and guidelines.

### A lack of communication infrastructure

Hospitals did not have working equipment and systems that enable health workers to call-for-help. There was a lack of hospital telephones and there are no alarm systems to activate when a health worker needs help. Available equipment were either broken or required spare parts such as batteries which were often not available.

“We don’t have communication equipment, it used to be there but ziliharibika zote (they all broke down)” *Nurse, Kenya*

“One time there was an alarm in the labor room, but I think it has problems or the battery died.” *Nurse, Tanzania*

Hospitals did not have specific communication infrastructure – personal phones were used instead. In some hospitals, calls were directed to an individual who had the necessary equipment and that person then communicated with those who could provide help, causing delays.

*“No, they can call overall hospital who will inform us, the call is first directed to hospital overall, then he/she call a person on site to inform about being needed”* Nurse, Tanzania

*“We do use our mobile phones to call the consultants, but it depends, because some of the consultants have their rules on communication…its challenging”* Nurse, Kenya

### Shortage of human resources

Hospitals did not have sufficient human resources to facilitate a smooth calling-for-help system. In some hospitals, at night there was only one doctor to serve the entire hospital. In many cases, there was simply no one to call-for-help. In some instances, when there were sufficient doctors available, there was only one nurse serving two wards making it difficult to recognize a deteriorating patient or to initiate or respond to a call-for-help on time:

*“There is one doctor who is general but there is also second on call who is at home but standby for emergency he/she will be called anytime of emergency, there is one nurse per ward, due to our shortages there is no way of having more than one nurse”* Doctor, Tanzania

*“So, you may need the help from the doctor and at that time he might have been called already at another ward”* Nurse, Tanzania

*“We sometimes have very serious nurses shortage, becomes difficult to move around”* Nurse, Kenya

*“It is sometimes difficult, there are times when I do manage two wards alone, and you find a situation where you have an emergency but you are alone, and it becomes difficult to shout for help as you become overwhelmed”* Nurse, Tanzania

*“We are few so it is not always easy to find help because you cannot leave a patient at bed critical and start chasing help”.* Nurse, Kenya

### Lack of routines and guidelines to systemize call for help

Hospitals lacked guiding documents that explain how the calling-for-help should be done, when it should happen, who should call and be called, and what should happen and how. Standardized routines for the process of calling-for-help did not exist. The interviews demonstrated that individual clinicians expected different routines and practices even when it comes to calling-for-help.

*“That is dependent on the consultant of course you know some consultants have their rules they are like, ‘Yes that is the one who has to call me,’ but some of the consultants are easy so you can easily just say let me call doctor so-and-so to come and consult in….”* Nurse, Kenya

*“Now it depends, some of the doctors will be around at the hospital, but some will be at ho”.me until you call them to come. And it depends on the severity of the problem, he may arrive on time or he might be late* Nurse, Tanzania

“*Calling-for-help efficiency has no one standard, in daytime is different as in night time, as day time there many people to help (health workers) while night is challenging”* Nurse, Tanzania

#### Theme 2 Calling-for-help processes: the calling-for-help processes are innovative and improvised

As the hospitals lack the structures required and do not have established standardized routines to guide the calling-for-help, participants described innovative strategies for the process of calling-for-help. Health workers used a variety of approaches and decided what to do on an improvised basis. The innovative, improvised processes can be divided into two categories: personally calling-for-help, and using others to call-for-help.

### Health workers personally calling-for-help

The chronic and severe shortage of human resources in healthcare in Tanzania and Kenya can result in a health worker being alone and finding themselves to be the only person around who can call-for-help. As well as attempting to provide care for the critically ill patient, the health worker is forced to call-for-help themselves. The participants described walking to other wards or nearby offices themselves to look for help, staying by the patient and shouting loudly to call-for-help, or using their personal mobile phones to contact someone who could come to help.

*“But at that moment I will also walk around other wards to see a nearby doctor who can help me while waiting for another doctor”* Nurse, Tanzania

*“Service provider will call loudly and shout, ‘Help!’ and any one close to there will run fast at the end of the day we will be a team”* Nurse, Tanzania

*“You do apply several approaches to get help, you may use mobile or hospital phone sometimes”* Nurse, Kenya

### Health workers use others to call-for-help

Participants reported that, at times, they turn to other people in the ward to call-for-help. This can be from other staff, from other in-patients, or from relatives or other care-takers of the patient or other patients.

*“She has to ask a patient who can walk to call other nurses or doctors in another ward, therefore it is difficult”* Doctor, Tanzania

*“There are moments where relatives become of great importance, we use them as messengers to run around and call other health workers to come and help”* Nurse, Tanzania

#### Theme 3 Calling-for-help outcomes: the help that is provided is not as requested

When health workers call for help, they expect it to fit their needs and arrive on time. However, the study participants described challenges with this, and the help they receive doesn’t always match what they asked for. At times the assistance was delayed – the person who needed to provide help did not arrive promptly, or busy phone lines hindered the communication of help***.*** At other times the help arrived, but was not the specific type of assistance they requested.

*“Because sometimes the phone is busy and you don’t get on time. So, delays of help is what I experience most of the time”.* Nurse, Tanzania

*“Sometimes they come on time, but sometimes they get late.”* Nurse, Tanzania

*“Not all time you will get help you requested, but you just have to adapt”* Nurse, Kenya

*“Sometimes also you may need a senior doctor, but because of challenges you may end up having your fellow nurse”* Nurse, Tanzania

## Discussion

Health workers in Tanzania and Kenya describe weak systems for calling-for-help when managing critically ill patients in hospitals. Three themes have been identified: 1) Calling-for-help structures: there are weak structures for calling-for-help; 2) Calling-for-help processes: the calling-for-help processes are innovative and improvised; and 3) Calling-for-help outcomes: the help that is provided is not as requested.

Adequate structures are required for managing patient deterioration [23, 24], patient safety [25] and in facilitating communication in health care teams [26]. Our study found that hospitals lack the structures for calling-for-help such as functional phones and alarm systems, which can be a large contributor to adverse clinical effects [26] and potentially leading to increased hospital mortality [27] as they prevent access to help. Additionally, hospitals have a shortage of human resources, a well-known issue in Kenyan and Tanzanian hospitals, that has been specifically shown to increase the risk of failing to rescue a patient from deterioration [28], which is the main aim of calling-for-help. While staff shortages may be an explanation for poor calling-for-help systems, it does not make them inevitable. Rather, calling-for-help systems are particularly important where there is shortage of staff to enable quick relocation and optimized outcomes of limited resources. Vincent et al. argued that calling-for-help as a part of care escalation process should be directed by guidelines and routines for smooth implementation [27], but the hospitals in our study lacked calling-for-help protocols. Guidelines can also improve the consistency of care [29, 30], and the described lack of consistency where different health workers behaved or practiced differently may impact quality of care. As Donabedian said: “a good structure increases the likelihood of good process, and good process increases the likelihood of good outcomes” [31].

Our study identified a lack of standardized processes for calling-for-help which led health workers to innovate when calling-for-help. Health workers were either personally calling-for-help by shouting, using their private mobile phones, physically looking for help outside the ward, or by using others like relatives and other patients to call-for-help. While some methods like involving patients [32, 33] may lead to adequate calling-for-help, improvised processes are, by their nature, non-standardized, and will vary in effectiveness. An improvised, ineffective process will impact the delivery of quality care [26] and risk negatively affecting patient outcomes [28]. Although meeting international standards of care may not be possible in low-resource settings without increased provision of resources, the absence of standardized, locally relevant processes for calling-for-help risks unnecessary harms.

Health workers reported not getting the type or quality of help they requested, and if it did arrive, the quality of help provided varied, possibly leading to missed care, adverse events or death [34]. A contributing factor is the absence of appropriate senior support and resources for higher level patient management which hinders the possibility of taking appropriate action [35]. Furthermore, it has been found that when help is requested, it is often delayed which can be hazardous when managing critically ill patients whose conditions require time-sensitive intervention [36]. Studies have shown prompt responses to calls for help can significantly improve the outcome of critically ill patients [37]. Therefore, improving the effectiveness of calling-for help mechanisms within hospitals, so that help is delivered promptly when requested, has the potential to improve health outcomes of critically ill patients.

**Fig. 2:**
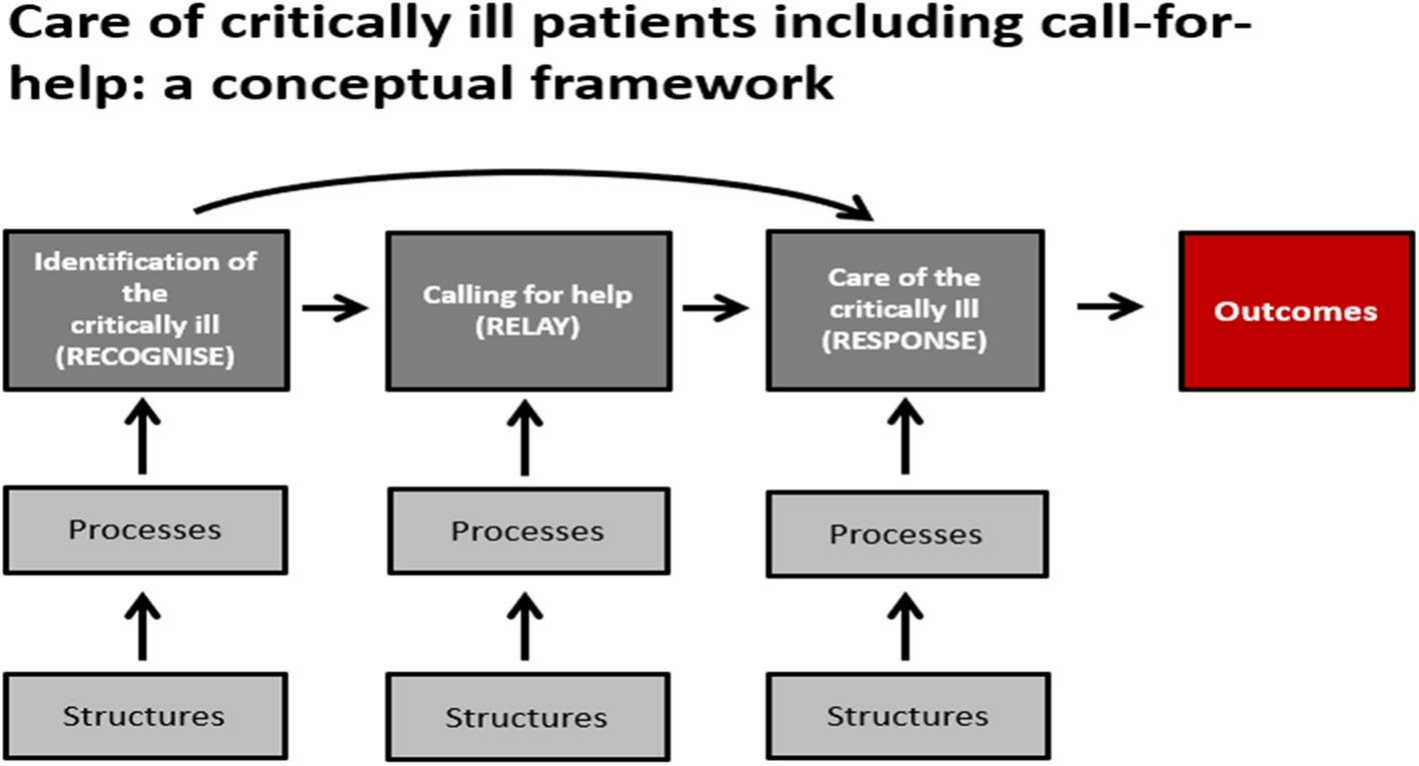
Including call-for-help in the Essential Emergency and Critical Care (EECC) conceptual framework for caring for critically ill patients (Modified from Schell 2021 [38]). The EECC Conceptual Framework by Schell and co-authors had only the first (identification) and second (Care) domains leading to Outcomes. We propose adding a Calling-for-help domain in between the other domains that can be an important function in health facilities for connecting identification and care. This brings the framework in-line with the model of ‘Recognize, Relay and Response’ proposed by Burke and co-authors (added to domain boxes in the Figure) [39]

In previous critical illness work, a conceptual framework was developed for the provision of effective care of the critically ill [38]. In the framework, the two described vital domains of care are *identification* of the critically ill and *care* of the critically ill. Our results suggest an addition to this framework—calling-for-help (Fig. 2). When a critically ill patient is identified, health workers may need to call for help from colleagues, and a system for this requires structures and processes.

Calling-for-help also has an influence on ‘failure-to-rescue’, a term used in some literature to refer to the inability to recognize and respond to a patient’s deteriorating condition in a timely and effective manner [39]. In many cases, failure-to-rescue occurs when warning signs of a patient’s deterioration are present, but healthcare providers fail to take appropriate action [39]. In the failure-to-rescue literature, the 3-steps of Recognize-Relay-Respond have been proposed as model of action. ‘Relay’ in this model is a synonym for calling-for-help [39] and as an integral component of preventing failure-to-rescue, hospitals should strengthen calling -for-help systems.

This study has several strengths. Firstly, we used a qualitative methodology that allowed us to explore the experiences of health workers in calling-for-help in some depth. Secondly, we used a purposive sampling strategy to recruit a diverse sample of participants, which helped us gain a broad range of perspectives on the research question. Lastly, we analyzed our data using the established Donabedian framework. However, the study also has several limitations. Firstly the study used a small sample size of hospitals which allowed for an in-depth exploration of the research question, but may limit the generalizability of the findings to other populations or contexts. Secondly, the study did not investigate cultural and systemic factors influencing health workers’ behavior when calling-for-help, particularly in relation to issues such as shame-and-blame or accountability.

## Conclusion

Calling-for-help when taking care of a critically ill patient is a necessary life-saving part of care. However, health-workers in Tanzanian and Kenyan hospitals experience significant challenges. Hospitals have weak structures for calling-for-help, the process is improvised, and the help that is provided is not as requested. These challenges likely cause delays and decrease safety and the quality of care, potentially resulting in unnecessary mortality and morbidity.

### Supplementary Information

Supplementary Material 1: Appendix 1. Thematic analysis.

Supplementary Material 2.

## Data Availability

The datasets used and/or analyzed during the current study are available from the corresponding author on reasonable request.
