# Supplementary material for: Health care workers’ experiences of calling-for-help when taking care of critically ill patients in hospitals in Tanzania and Kenya
Source: BMC Health Serv Res. 2024 Jul 17;24:821. doi: 10.1186/s12913-024-11254-y (PMC11253331; doi:10.1186/s12913-024-11254-y)
Supplement: Supplementary file 1 — Supplementary Material 1: Appendix 1. Thematic analysis. [file 12913_2024_11254_MOESM1_ESM.docx]

Appendix 1: Thematic analysis

| **Initial codes** | **Categories** | **Themes** |
| --- | --- | --- |
| There are not enough communication devices | Lack of functional communication infrastructure | **There is a lack of functioning structures for calling-for-help** |
| Communication devices broken |  |  |
| There is not a standard system for communication |  |  |
| Centralized communication model delays communication |  |  |
| There is not enough staff | Insufficient human resources |  |
| Doctors are staying far from hospital |  |  |
| Low nurses: patient ratios |  |  |
| There are no guidelines for calling-for-help | Lack of routines and guidelines |  |
| Model of response depends on the personality of the health worker |  |  |
| Effectiveness of response depends on the time the event happens |  |  |
| Walking around looking for help | Health workers personally calling-for-help | **The calling-for-help processes are innovative and improvised** |
| Shouting loud asking for help |  |  |
| Calling using personal mobile phone |  |  |
| Asking relatives to assist getting help | Health workers use others to call-for help |  |
| Asking patients to assist getting help |  |  |
| Help arrives late | Delayed Assistance | **The help that is provided is not as requested** |
| Getting help in time depends on what time (day/night) the call is made |  |  |
| Getting help of a lower level than requested | Help received is not as requested |  |
| Poor quality help |  |  |
