# Supplementary material for: Health care workers’ experiences of calling-for-help when taking care of critically ill patients in hospitals in Tanzania and Kenya
Source: BMC Health Serv Res. 2024 Jul 17;24:821. doi: 10.1186/s12913-024-11254-y (PMC11253331; doi:10.1186/s12913-024-11254-y)
Supplement: Supplementary file 2 — Supplementary Material 2. [file 12913_2024_11254_MOESM2_ESM.docx]

**CALLING FOR HELP - INTERVIEW GUIDE FOR HEALTHCARE WORKERS**

**Introduction**

During their stay in hospital, the condition of some patients becomes life-threatening, with a high risk of death. These patients are challenging for a health worker to manage alone, and it is common practice to **call for help** from other health workers, either from senior staff or from health workers specialised in managing such patients.

We would like to know about your experiences of calling-for-help when taking care of such “critically ill” patients with life-threatening conditions.

**PART 1: Consent for recording**

*Before we begin, please can you confirm that you consent to having this interview recorded in line with the information sheet shared?* *Everything you say is confidential, the recorded data will not be shared with anyone outside the research team and the data will be anonymized.*

**PART 2: Introduction and basic information**

*Thank you for agreeing to participate in this research. Before we begin, I would just like to emphasize again that this is not an inspection or an audit – and no information specific to this hospital will be fed back to your managers. We are just interested in understanding more about care of critically ill patients in hospitals. So please feel at ease to be as open as possible with your responses.*

| **Date for interview** | **Day** [ ] [ ] **Month**  [ ] [ ] **Year** [ ] [ ] [ ] [ ] |
| --- | --- |
| **Interviewer name** |  |
| **Region Name** |  |
| **District Name** |  |
| **CODE** E.g. HCW_TZ_H1_001_01 |  |
| **GENERAL INFORMATION ABOUT RESPONDENT** | |
| **Gender** | 1. Male … 01 2. Female… 02 |
| **Age** |  |
| **Job Title** |  |
| **Name of hospital** |  |
| **Name of department** |  |
| **Highest educational qualification achieved?** |  |
| **What kind of medical training do you have?** |  |
| **How many years have you been qualified for this post?** |  |
| **How many years have you been working at this hospital?** |  |

**NOTE TO INTERVIEWER: PROVIDE OUR DEFINITION OF CRITICAL ILLNESS AND CALLING FOR HELP EXPLAIN THAT THE INTERVIEW QUESTIONS ARE BASED ON THIS DEFINITIONS.**

***Critical Illness*** *is any patient with a life-threatening condition.*

***Calling-for-help*** *is the process of asking (or trying to ask) for assistance from colleagues when looking after a critically ill patient.*

**When you have a need to call for help, please can you describe how this is done and what are your experiences of doing it?**

Prompts:

How do you send out your call?

Which means do you use to call for help?

Is there a formal protocol / routine in place for calling-for-help?

Are there written guidelines for how to call-for-help?

Is it clear who you should call, and how to call them?

Who does the calling-for-help?

Do you have enough staff available to call-for-help?

Do you have any communication system that you can use?

What is the condition of the communication system?

If there is no system, how does communication happen?

Is it always the same way that is used, or are there different ways?

Do you ever call-for-help using your private phone, using patient relatives, other patients, shouting loudly, other?

Are you satisfied with the way of calling-for-help?

What are the good aspects about calling-for-help in your hospital?

What could be improved?

**When you call-for-help, what response do you get?**

Prompts

Are senior staff available in the hospital to respond to the call for help?

Which cadres and types of staff?

Are there differences between night and day shifts? Weekends?

Do you get help?

How long before you get help?

Do you think you get help on time? Why /Why not?

Do you get help via the phone, or physically?

Are you satisfied with the help you receive?

What are the good aspects about the help you receive in your hospital?

What could be improved?

***Thank you very much for your time.***
